# The endolysosomal system in conventional and unconventional protein secretion

**DOI:** 10.1083/jcb.202404152

**Published:** 2024-08-12

**Authors:** Eloïse Néel, Marioara Chiritoiu-Butnaru, William Fargues, Morgane Denus, Maëlle Colladant, Aurore Filaquier, Sarah E. Stewart, Sylvain Lehmann, Chiara Zurzolo, David C. Rubinsztein, Philippe Marin, Marie-Laure Parmentier, Julien Villeneuve

**Affiliations:** 1 Institute of Functional Genomics, University of Montpellier, CNRS, INSERM, Montpellier, France; 2 Institute of Biochemistry of the Romanian Academy, Bucharest, Romania; 3 Department of Biochemistry and Chemistry, La Trobe Institute for Molecular Science, La Trobe University, Melbourne, Australia; 4 Laboratoire de Biochimie-Protéomique Clinique–Plateforme de Protéomique Clinique, Université de Montpellier, Institute for Regenerative Medicine and Biotherapy Centre Hospitalier Universitaire de Montpellier, Institute for Neurosciences of Montpellier INSERM, Montpellier, France; 5 Unité de Trafic Membranaire et Pathogenèse, Institut Pasteur, UMR3691 CNRS, Paris, France; 6 Department of Medical Genetics, Cambridge Institute for Medical Research, University of Cambridge, Cambridge, UK; 7 UK Dementia Research Institute, Cambridge, UK

## Abstract

Most secreted proteins are transported through the “conventional” endoplasmic reticulum–Golgi apparatus exocytic route for their delivery to the cell surface and release into the extracellular space. Nonetheless, formative discoveries have underscored the existence of alternative or “unconventional” secretory routes, which play a crucial role in exporting a diverse array of cytosolic proteins outside the cell in response to intrinsic demands, external cues, and environmental changes. In this context, lysosomes emerge as dynamic organelles positioned at the crossroads of multiple intracellular trafficking pathways, endowed with the capacity to fuse with the plasma membrane and recognized for their key role in both conventional and unconventional protein secretion. The recent recognition of lysosomal transport and exocytosis in the unconventional secretion of cargo proteins provides new and promising insights into our understanding of numerous physiological processes.

## Teaser

Néel et al. review how endolysosomes, positioned at the crossroads of multiple intracellular trafficking pathways, play critical roles in conventional and unconventional protein secretion.

## Introduction

Our understanding of the basic machinery governing intracellular transport via the conventional endoplasmic reticulum (ER)–Golgi secretory pathway has recently been expanded by discoveries highlighting alternative mechanisms that drive secretion. These unconventional secretory routes involve multiple cellular processes facilitating the active and selective release of cytosolic proteins lacking an N-terminal signal sequence for ER entry (Filaquier et al., 2022; Zhang and Schekman, 2013). Unexpectedly, lysosomes have emerged as multifaceted intracellular compartments where specific cargo proteins, with or without N-terminal signal sequence, can converge before being released into the extracellular space by lysosomal exocytosis (Tancini et al., 2020; Filaquier et al., 2022).

The diverse array of lysosomal functions relies on the cooperation of an extensive repertoire of molecular factors, including luminal hydrolases, integral lysosomal membrane proteins (LMPs), and lysosome-associated proteins. Approximately 60 acid hydrolases are actively involved in the degradation and recycling of macromolecules delivered to lysosomes, and ∼120 LMPs sustain other lysosome properties, such as acidification, nutrient and ion transport, protein import from the cytosol, and lysosome fusion and interactions with other intracellular compartments. In addition, lysosome-associated proteins are dynamically recruited from the cytosol to the lysosomal surface, making lysosomes central hubs for nutrient and nucleic acid sensing and platforms for multiple trafficking and signaling pathways critical for regulating energy metabolism and adapting to cellular stress (Chapel et al., 2013; Ballabio and Bonifacino, 2020; Perera and Zoncu, 2016). Thus, lysosomes are no longer only considered as terminal degradative organelles but as versatile compartments with diverse functions essential for the maintenance of cellular homeostasis (Hämälistö et al., 2024; Roney et al., 2022). Furthermore, lysosomes are highly dynamic organelles that can fuse with the plasma membrane (PM) and thereby release their contents into the extracellular milieu, contributing for instance to PM integrity, extracellular matrix (ECM) remodeling, and defense against pathogens (Tancini et al., 2020; Blott and Griffiths, 2002; Andrews, 2000; Villeneuve et al., 2018).

**Figure 1.**
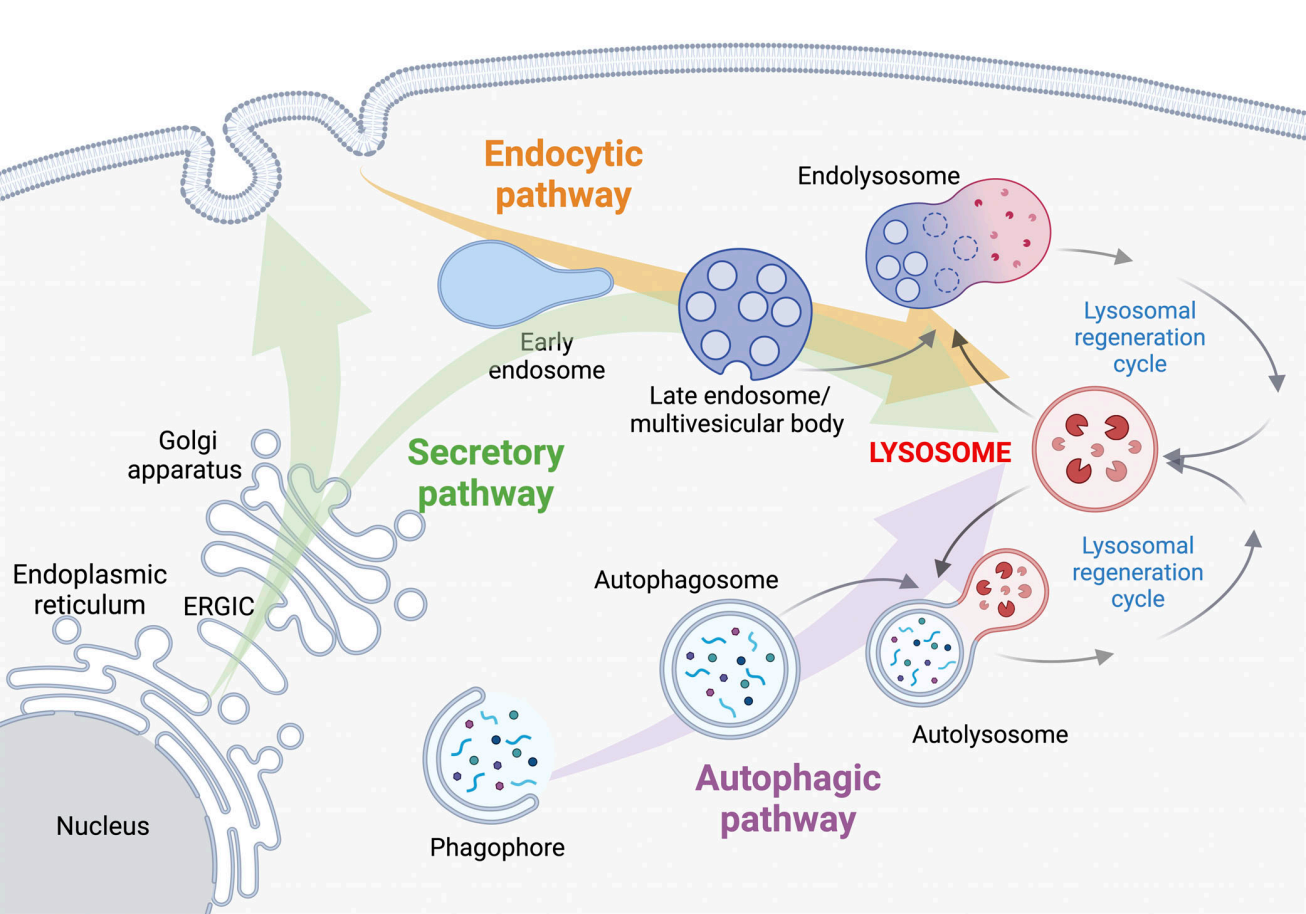
**Lysosomes are at the crossroad of several intracellular trafficking pathways.** Newly synthesized lysosomal hydrolases and LMPs contain an N-terminal hydrophobic signal sequence or transmembrane domains that allow their targeting and insertion into the ER. Once transported through the Golgi apparatus, lysosomal proteins are then subjected to post-translational modifications including the addition of mannose-6-phosphate (M6P) residues. M6P residues are essential for protein sorting at the TGN, where lysosomal proteins are packaged into clathrin-coated intermediates and diverted from the biosynthetic secretory pathway to the endocytic pathway for delivery to lysosomes. Post-Golgi clathrin-coated carriers are mainly targeted directly to the endolysosomal pathway (Polishchuk et al., 2006). This pathway relies on coordinated membrane fluxes involving organelle maturation and membrane fission/fusion events (Huotari and Helenius, 2011). In addition to the well-characterized direct transport to lysosomes, several newly synthesized LMPs follow an indirect pathway. This pathway entails the transport of LMPs to the PM, their subsequent internalization into early endosomes, and delivery to LEs and lysosomes (Braun et al., 1989). By combining innovative methods like the “retention using selective hooks” system with high-resolution live-cell imaging, studies revealed the spatiotemporal dynamics of these distinct pathways (Chen et al., 2017). The development of pooled genome-wide CRISPR screens designed to identify factors involved in protein transport and secretion holds promise for addressing these crucial issues (Bassaganyas et al., 2019; Popa et al., 2019). In addition to functional factors and extracellular substrates transported to lysosomes via the secretory and endocytic pathways, cytoplasmic material is targeted to lysosomes through sequestration by macroautophagy. In the autophagic process, cytoplasmic substrates are initially sequestered within a cup-shaped double membrane structure known as the phagophore, which upon expansion and complete closure forms the autophagosome. Autophagosomes are then transported along the microtubules and ultimately fuse with lysosomes (Bento et al., 2016). Along the endocytic and autophagic pathways, the fusion of lysosomes with LE/MVBs and autophagosomes forms endolysosomes and autolysosomes, respectively. From these hybrid compartments, lysosomes or terminal/storage lysosomes are reformed via lysosomal regeneration cycles, through processes of tubulation, maturation, and content condensation (Yang and Wang, 2021). In this review, the terms “lysosomes,” “endolysosomes,” or “LE/lysosomes” are used alternatively due to uncertainty in the cited references regarding the exact nature of the described organelles. Illustration created with Biorender.

A notable feature of lysosomes is their unique position at the intersection of various intracellular trafficking pathways, including autophagic, endocytic, and biosynthetic secretory routes (Fig. 1). These intricate interconnections, coupled with the dynamic nature of lysosomes and their exocytic capabilities, endow this organelle with essential roles in the secretion of cargo proteins transported by both conventional and unconventional secretory routes. In this review, we will explore emerging evidence establishing lysosomes as central trafficking stations in various secretory pathways and examine how lysosomal properties can be modulated converting lysosomes into secretory compartments.

## Lysosomes as transport carriers for both conventional and unconventional protein secretion

### Lysosomes are transport carriers for the secretion of signal sequence–containing cargo proteins

In eukaryotes, most secreted and PM proteins follow the conventional secretory pathway in which they are first targeted and inserted into the ER membrane via their N-terminal hydrophobic signal sequences or transmembrane domains (Rapoport, 2007). Then, proteins progress through the Golgi apparatus and are subsequently transported from the trans-Golgi network (TGN) to the PM (Schekman, 2008). A distinction is commonly made between constitutive and regulated secretion. Constitutive secretion relies on transport carriers that continuously shuttle from the TGN to the PM, yet their diversity and biogenesis mechanisms remain insufficiently understood (Chen et al., 2017; De Matteis and Luini, 2008; Stalder and Gershlick, 2020). This process occurs in most cell types and contributes to the release of enzymes, growth factors, and ECM components, among others (Parchure and von Blume, 2023; Holcomb et al., 1988; Bossard et al., 2007). In regulated secretion, cargo proteins are sorted at the TGN into specialized secretory granules that fuse with the PM in a controlled manner upon stimulation by external factors. This process is characteristic of specialized cell types such as pancreatic β cells, endothelial cells, intestinal goblet cells, and neuroendocrine cells, to rapidly mobilize and export insulin, von Willebrand factor, mucin, and chromogranin, respectively (Burgoyne and Morgan, 2003; Davis and Dickey, 2008; Bauerfeind and Huttner, 1993).

In addition to post-Golgi carriers involved in constitutive and regulated secretion, it is now established that lysosomes derived from the endocytic system (Fig. 1) can also fuse with the PM and release their contents into the extracellular milieu (Andrews, 2000; Tancini et al., 2020). Lysosomal exocytosis triggered by external cues was initially thought to be restricted to cell type–specific organelles known as specialized lysosomes or lysosome-related organelles (LROs). These were identified on the basis of compositional and physiological features shared with standard degradative lysosomes. LROs include lytic granules in cytotoxic T lymphocytes and natural killer cells (Blott and Griffiths, 2002), major histocompatibility complex class II compartments in macrophages, dendritic cells, and B lymphocytes (Harding, 1996); basophil granules in basophils and mast cells (Schwartz and Austen, 1980); azurophil granules in neutrophils (Kjeldsen et al., 1993); dense granules in blood platelets (Van Oost et al., 1985); and melanosomes in melanocytes (Orlow, 1995). Reminiscent of standard degradative lysosomes, these organelles are acidic compartments with membranes derived primarily from the endocytic system. Most LROs contain enzymes and LMPs essential for substrate degradation and recycling. They also package newly synthesized secreted proteins based on their specialized biological function. Similar to standard lysosomes, structural and functional factors delivered to specialized lysosomes are transported through the ER–Golgi secretory pathway, either directly or indirectly after recognizing a tyrosine or dileucine-based signal in the amino acid sequence of cargoes or cargo receptors (Dell’Angelica et al., 2000). While both standard and specialized lysosomes coexist in some cell types, such as in melanocytes and blood platelets, lytic granules are the major lysosomal structures in cytotoxic T lymphocytes (Luzio et al., 2014). In this context, it is important to distinguish lysosomes and specialized lysosomes from other structures containing electron-dense proteins, often referred to as LROs. One example is Weibel-Palade bodies in endothelial cells, which directly originate from the TGN but not from the endocytic pathway, in contrast with standard and specialized lysosomes (Raposo et al., 2007). The close relationship between standard and specialized lysosomes is underscored by observations in some human autosomal recessive disorders, such as Chediak-Higashi, Hermansky-Pudlak, and Griscelli’s syndromes, which share common clinical features, including varying degrees of hypopigmentation due to impaired melanosome secretion, prolonged bleeding due to defects in dense granule secretion by blood platelets, and immunologic deficiencies due to impaired secretion of various granules by immune cells (Bowman et al., 2019; Bonifacino, 2004). At the cellular level, patients with Chediak-Higashi syndrome exhibit abnormal morphology and numbers of lysosomes and LROs (De Chadarévian, 2011; Durchfort et al., 2012). These genetic disorders, characterized by defects in the biogenesis of both standard and specialized lysosomes and governed by numerous common molecular determinants, further support the common organellar lineage of these structures.

In addition to LROs, the presence of lysosomes, which exhibit exocytosis, was then recognized not only in lower eukaryotes, such as *Dictyostelium discoideum*, *Leishmania donovani*, and *Tetrahymena pyriformis* (Dimond et al., 1981; Gottlieb and Dwyer, 1981; Müller, 1972), but also in various cell types from higher eukaryotes and humans, including hepatocytes and pancreatic acinar cells, which release lysosomal hydrolases to promote digestion, and osteoclasts to promote bone resorption (LeSage et al., 1993; Mostov and Werb, 1997; Gross et al., 1985). In cancer cells, the release of lysosomal hydrolases promotes tumorigenesis, invasion, and metastasis (Bian et al., 2016; Sevenich and Joyce, 2014).

Subsequently, regulated exocytosis of lysosomes has been observed in most cell types. In particular, studies indicate that an increase in intracellular Ca^2+^ concentration triggers lysosomal exocytosis in a temperature- and ATP-dependent manner in fibroblasts, epithelial cells, and myoblasts. In these experiments, lysosomal exocytosis was monitored by the release of fluid phase tracers previously loaded into lysosomes, the increased expression of cell surface LMPs, and the release of lysosomal enzymes, such as the processed form of cathepsin D (Rodríguez et al., 1997; Andrews, 2000). Similar to the Ca^2+^-regulated exocytosis of post-Golgi secretory granules in specialized secretory cells, lysosomal exocytosis represents a ubiquitous form of Ca^2+^-regulated exocytosis. Further studies have revealed that sequential Ca^2+^-regulated lysosomal exocytosis and massive endocytosis cooperate to maintain PM integrity in wounded cells (Reddy et al., 2001; Jaiswal et al., 2002; Hilgemann et al., 2013). In addition to providing the endomembrane necessary for PM resealing, lysosomal exocytosis also mobilizes lysosomal hydrolases to the cell surface, playing a critical role in PM repair. For example, acid sphingomyelinase, released by lysosomal exocytosis, can convert sphingomyelin to ceramide on the outer leaflet of the PM. These ceramide-enriched domains on the cell surface promote lipid bilayer invagination and rapid endosome formation, contributing to the removal of PM lesions (Tam et al., 2010; Schuchman, 2010). Other secreted lysosomal cysteine proteases have been implicated in this process by facilitating acid sphingomyelinase access to the PM through ECM degradation (Castro-Gomes et al., 2016).

**Figure 2.**
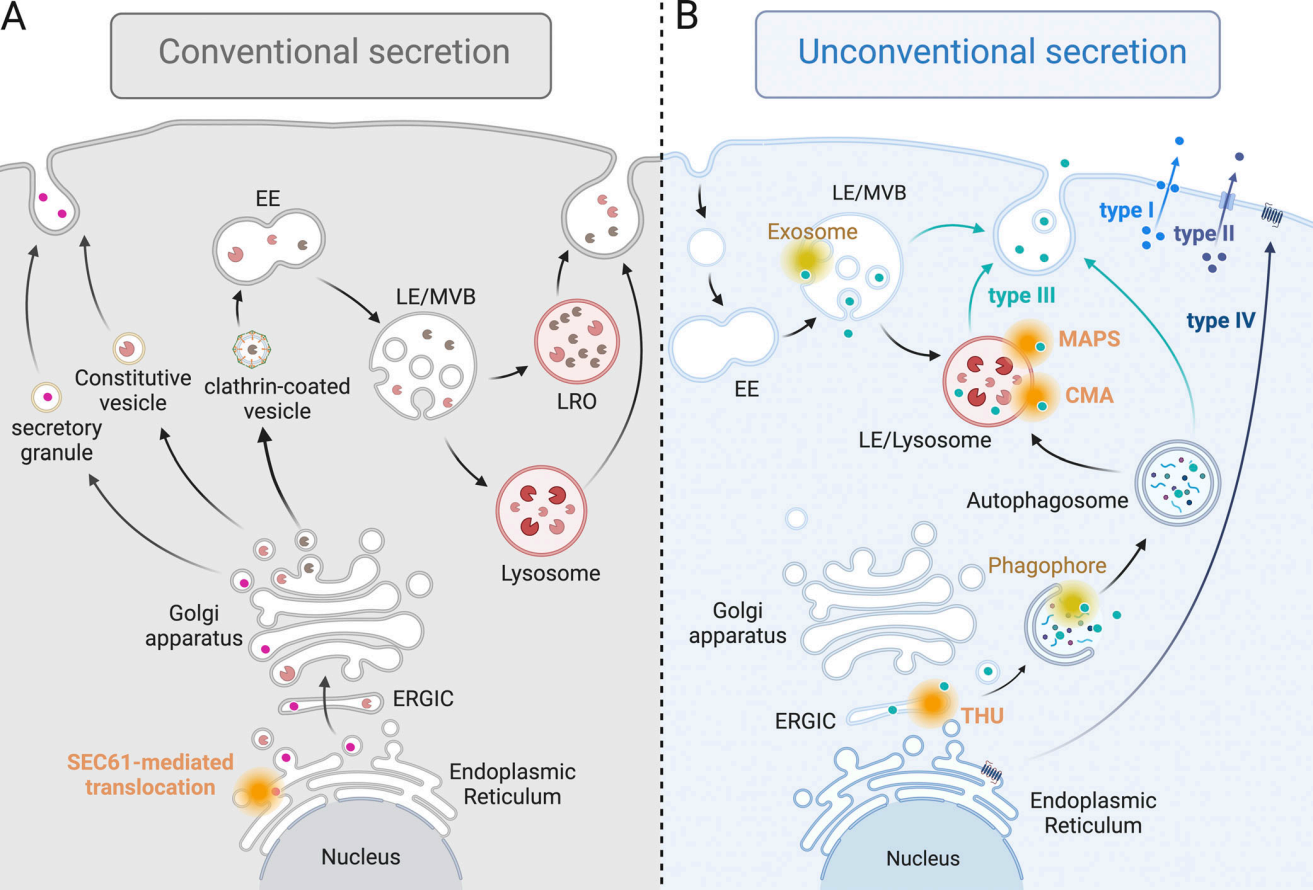
**Conventional and unconventional protein secretion. (A)** Proteins with a signal peptide reach their final destination (the extracellular space, the PM, or lysosomes) after transport along the conventional or biosynthetic secretory pathway. Briefly, cargo proteins are inserted into the ER via the SEC61 channel-forming translocon complex and then sorted by the use of COPII-coated vesicles to reach the Golgi apparatus. At the level of the trans-Golgi network, constitutive secretory proteins such as growth factors and ECM components are packaged into constitutive vesicles, whereas specific proteins such as insulin and mucin are packaged into secretory granules according to cell type. Structural and functional lysosomal proteins are packaged into clathrin-coated vesicles and transported to the endolysosomal system. Specialized lysosomes or LROs and standard lysosomes can then behave as secretory vesicles, releasing cargo proteins containing a signal sequence as post-Golgi carriers. **(B)** Proteins without a signal peptide can be secreted via unconventional protein secretion (UcPS), independent of the conventional secretory pathway. In type I and II UcPS, cytosolic proteins are secreted by direct translocation across the PM through protein channels and ABC transporters, respectively. In type III UcPS, cargo proteins are incorporated into membrane intermediates through protein channels (orange) or membrane remodeling (yellow) that define multiple entry gates along the biosynthetic secretory, endocytic, or autophagic pathways. In type IV UcPS, integral membrane proteins located in the ER are transported to the PM independently of the Golgi apparatus. CMA: Chaperone-mediated autophagy; EE: Early endosome; ERGIC: Endoplasmic reticulum–Golgi intermediate compartment; LE: Late endosome; MAPS: Misfolded-associated protein secretion; MVB: Multivesicular body; THU: TMED10-channeled UcPS. The figure was created with Biorender.

In summary, similar to post-Golgi carriers, lysosomes also behave as secretory vesicles that fuse with the PM and release their contents into the extracellular milieu in a regulated manner (Fig. 2 A). This underscores the critical role of lysosomes as organelles that regulate diverse cellular and physiological functions, including metabolism, innate and adaptive immunity, thrombosis and hemostasis, ECM remodeling, maintenance of PM integrity, and cell survival.

### Lysosomes are transport carriers for the unconventional secretion of cargo proteins lacking a signal sequence

An increasing number of cytosolic proteins lacking a signal sequence for ER entry have been shown to be actively and selectively exported from eukaryotic cells. Remarkably, this process is independent of the highly conserved ER–Golgi secretory pathway. Protein families transported via unconventional secretory pathways include inflammatory cytokines, annexins, heat shock proteins, lipid chaperones, antioxidant enzymes, and misfolded proteins, among others (Rabouille, 2017; Zhang and Schekman, 2013; Filaquier et al., 2022). In recent years, there has been extensive documentation of the extracellular functions of these proteins and the impact of disrupting their secretion in various diseases (Cao et al., 2013; Radisky et al., 2009; Sitia and Rubartelli, 2020). Despite the identification of numerous alternative routes for the export of cytosolic proteins, the molecular trafficking machinery remains poorly understood in most cases (Fig. 2 B). In type I unconventional protein secretion (UcPS), cargo proteins are directly translocated across the PM. Proposed mechanisms include the formation of pores by gasdermin D for the release of interleukin-1β (IL-1β) in macrophages (Evavold et al., 2018), or the formation of pores through the oligomerization of fibroblast growth factor-2 (FGF-2), followed by its insertion into the PM, a necessary step for its secretion (Steringer et al., 2017). As with FGF-2 secretion, self-translocation has been reported for HIV-Tat secretion (Zeitler et al., 2015). Lipidated proteins, such as the farnesylated pheromone m- and α-factors in yeast, are also directly translocated across the PM. In this case, translocation is facilitated by ATP-binding cassette (ABC) transporters, and they are referred to as type II UcPS cargoes (Rabouille et al., 2012). In addition to type I and II UcPS, cytosolic proteins can be secreted into the extracellular milieu after transport through intermediate compartments derived from autophagic membranes, endosomes, multivesicular bodies (MVBs), or lysosomes that release their contents after exocytosis. This form of transport, termed type III UcPS, involves multiple membranes and protein trafficking pathways (Rabouille, 2017; Zhang and Schekman, 2013; Filaquier et al., 2022). Given the unique position of lysosomes at the intersection of multiple intracellular trafficking pathways, as discussed below, cytosolic cargo proteins trafficked by type III UcPS may converge to lysosomes before their release into the extracellular space (Fig. 2 B). In type IV UcPS or Golgi bypass, observed under conditions such as ER stress or after inhibition of the conventional secretory pathway, integral membrane proteins located in the ER are transported to the PM independently of the Golgi apparatus (Gee et al., 2018) (Fig. 2 B).

#### Membrane remodeling for type III UcPS

In type III UcPS, cytosolic material and cargo proteins can be incorporated into intermediate compartments through membrane remodeling. Autophagy allows the engulfment and sequestration of cytosolic material via autophagosome biogenesis. Genetic studies, initially performed in yeast and subsequently extended to mammalian cells, have shown that evolutionarily conserved autophagy-related genes (*ATG*) are required for the secretion of cytoplasmic substrates. These include the acyl-CoA-binding protein Acb1, inflammatory mediators, toxic protein aggregates or aggregation-prone proteins, and bacterial and viral pathogens (Duran et al., 2010; Dupont et al., 2011; Ejlerskov et al., 2013; Gerstenmaier et al., 2015). While the concept of secretory autophagy has recently gained attention, the direct involvement of autophagosomes in UcPS has not been clearly established. Autophagosomes have been proposed to deviate from their usual fusion with lysosomes and instead directly fuse with the PM in response to lysosomal damage. This rerouting was hypothesized to occur through the mobilization of dedicated soluble N-ethylmaleimide-sensitive-factor attachment protein receptors (SNAREs), in combination with a specialized tripartite motif containing (TRIM) cargo receptor (Kimura et al., 2017). Although several studies indicate that the *ATG* gene-dependent UcPS involves cytosolic cargo surrounded by an LC3-positive double membrane structure, a hallmark of autophagosomes, there is no conclusive evidence for direct fusion of autophagosomes with the PM (Dupont et al., 2011; Zhang et al., 2015; Ejlerskov et al., 2013). Instead, it is conceivable that autophagosomes might fuse with downstream membrane intermediates such as MVBs or lysosomes, which subsequently release their contents into the extracellular milieu after exocytosis (Ejlerskov et al., 2013; Baixauli et al., 2014; Takenouchi et al., 2009; Jeppesen et al., 2019). Alternatively, the autophagy machinery may intersect and influence other protein trafficking pathways and secretion events, as recently reviewed (Deretic et al., 2012; Levine and Kroemer, 2019; New and Thomas, 2019). For example, autophagy-deficient osteoclasts and mast cells exhibit altered secretion of lysosomal contents, leading to impaired bone resorption and compromised immune responses, respectively (DeSelm et al., 2011; Ushio et al., 2011). Also, while ATG-related proteins may be required for the early stages of the biogenesis of compartments for unconventional secretion (CUPS) in yeast, it is crucial to note that CUPS, which are critical for Acb1 secretion, are distinct from autophagosomes (Cruz-Garcia et al., 2014; Curwin et al., 2016). Whether autophagosomes can fuse directly with the PM or whether they act as vesicular intermediates that fuse with downstream vesicular compartments (e.g., MVBs or lysosomes) remains to be elucidated.

Another known membrane remodeling pathway for type III UcPS is via extracellular vesicles. These vesicles can be shed from the PM (known as microvesicles or ectosomes) or generated within the endolysosomal system (known as exosomes) (Meldolesi, 2022). Within the endolysosomal system, cytosolic proteins are sequestered by inward invagination of the membrane in late endosome (LE), followed by the budding and release of small vesicles into the lumen of the organelle, to ultimately form MVBs (Klumperman and Raposo, 2014; Frankel and Audhya, 2018). The endosomal sorting complex required for transport proteins plays a key role in this process (Remec Pavlin and Hurley, 2020). The resulting MVBs can then fuse with the PM, leading to the release of exosomes into the extracellular milieu. A critical process appears to be the conversion of phosphatidylinositol-3-phosphate (PI(3)P) to PI(4)P on the membrane of MVBs, which promotes the recruitment of the exocyst that directs MVBs for fusion with the PM (Liu et al., 2023). Alternatively, in adipocytes and B cells, exosomes formed along the endolysosomal pathway can also be released by lysosomal exocytosis and contribute to adipogenesis (Kim et al., 2019) and adaptive immune responses (Hämälistö et al., 2024), respectively.

#### Protein translocation for type III UcPS

In addition to pathways involving membrane remodeling to engulf cytosolic material, in type III UcPS, cargo proteins can also be translocated from the cytosol into intermediate compartments by dedicated transporters or channels.

A well-recognized mechanism for the selective degradation of intracellular components is chaperone-mediated autophagy (CMA), which enables the delivery of cytosolic proteins to the lysosomal surface and their subsequent translocation into the lysosomal lumen. In CMA, substrate recognition selectivity is mediated by a canonical KFERQ-like motif found in ∼40% of proteins in the mammalian proteome (Dice, 1990). In addition, posttranslational modifications, including phosphorylation or acetylation, modify the properties of alternative residues that contribute to the generation of this signal recognition motif or facilitate conformational changes that expose or mask this pentapeptide motif (Kaushik and Cuervo, 2016, 2018; Quintavalle et al., 2014). In CMA, the cytosolic chaperone HSC70 directly binds to the KFERQ-like motif and targets the protein to lysosomes (Chiang et al., 1989). It has been proposed that substrate translocation into lysosomes is mediated by the multimerization of LAMP2A, forming a 700 kDa translocation protein complex (Bandyopadhyay et al., 2010). However, whether LAMP2A forms a conducting channel for translocation remains to be confirmed by means of structural analysis and in vitro reconstitution approaches. Initially characterized as a selective degradation pathway critical for protein quality control, CMA has also been implicated in the presentation of cytoplasmic autoantigens at the cell surface (Zhou et al., 2005). In addition, studies suggest that IL-1β, which has three KFERQ-like motifs in its amino acid sequence, may be released from cells after translocation into lysosomes by a mechanism similar to CMA (Andrei et al., 1999; Zhang et al., 2015; Semino et al., 2018; Sitia and Rubartelli, 2020).

Unlike CMA, a process known as misfolded-associated protein secretion (MAPS) facilitates the translocation of misfolded cytosolic proteins into LE/lysosomes, followed by their exocytosis, when other protein quality control pathways, such as proteasomal degradation, fail to prevent excessive accumulation of aberrant polypeptides. Notable substrates for MAPS include cytosolic proteins associated with neurodegenerative diseases, such as Tau, ataxin-3, TDP43, and α-synuclein (Fontaine et al., 2016; Ye, 2018; Lee et al., 2016, 2018; Xu et al., 2018). When expressed in HEK293T cells, these misfolded-prone proteins are recognized by the ER-associated deubiquitinase USP19 and subsequently transferred to a complex formed by the chaperone HSC70 and its co-chaperone DnaJ heat shock protein member C5 (DNAJC5), which is anchored to endolysosomal compartments after palmitoylation (Xu et al., 2018; Wu et al., 2023; Lee et al., 2018). The function of DNAJC5 in α-synuclein UcPS was recently reconstituted in a variety of cells, including neurons, demonstrating the critical role of palmitoylated DNAJC5 in high-order oligomeric forms in α-synuclein UcPS (Wu et al., 2023). This study also reported that DNAJC5, together with α-synuclein, localizes and is translocated into the endolysosomal compartments, which presumably represent intermediates in α-synuclein secretion. In line with these observations, the authors also showed that bafilomycin A1, a lysosomal ATPase inhibitor known to promote secretion of MVBs and lysosomal contents (Cashikar and Hanson, 2019; Tapper and Sundler, 1995), promotes α-synuclein secretion. Of particular interest, the secreted α-synuclein mediated by palmitoylated DNAJC5 oligomers is mainly released into the extracellular space as soluble monomeric forms not enclosed within extracellular vesicles, whereas DNAJC5 is present in both soluble and extracellular vesicle-bound forms. The translocation machinery responsible for the transfer of misfolded proteins during MAPS has not been identified but does not appear to involve LAMP2A, as seen in CMA. The subsequent fusion of vesicular intermediates with the PM has been shown to be promoted by the SNAREs VAMP7, VAMP8, and SNAP-23, leading to the release of misfolded proteins from the cell (Ye, 2018; Fontaine et al., 2016; Lee et al., 2016, 2018). A recent study suggested that instead of using mainly endolysosomal compartments as vesicular intermediates, substrates exported from cells through the MAPS pathway and in a DNAJC5-dependent manner may be trafficked through an alternative vesicular intermediate (Lee et al., 2023). This compartment, located in the perinuclear area, near the Golgi apparatus, would be the equivalent of CUPS, identified in yeast as a critical compartment for UcPS (Lee et al., 2022). However, its exact nature and whether it is formed from Golgi and endosomal membranes like CUPS in yeast remains to be elucidated. It will also be important to establish whether these different vesicular intermediates, e.g., endolysosomes or CUPS, can be differentially mobilized for secretion of MAPS substrates, depending on cellular stress or experimental culture conditions. Although MAPS and CMA share common features, such as the involvement of chaperones that target cytosolic proteins to the LE-lysosomal compartment for translocation, these two pathways also exhibit important differences. In contrast to CMA, cargo recognition in MAPS is not mediated by a specific pentapeptide motif, but rather relies on exposed hydrophobic segments that characterize the misfolded state of protein substrates. Furthermore, as mentioned above, protein translocation into LE/lysosomes in MAPS does not require LAMP2 or cargo unfolding, in contrast to CMA (Lee et al., 2018; Ye, 2018). By exporting misfolded cytosolic proteins to the extracellular milieu using LE lysosomes as transport carriers, the MAPS pathway provides an additional protein quality control essential for maintaining homeostasis during proteotoxic stress.

It is likely that additional transporters or protein channels that facilitate cargo translocation, present at various membrane intermediates converging toward secretory compartments, remain to be uncovered and fully characterized. A recent study identified a novel protein translocation pathway called THU (TMED10-channeled UcPS) and demonstrated its ability to allow the entry of multiple cytosolic cargos into vesicular intermediates for UcPS (Zhang et al., 2020). The proposed model relies on the oligomerization of the transmembrane emp24 domain-containing protein 10 (TMED10) to form a channel that facilitates the translocation of cargo proteins into a specialized subcompartment of the ER–Golgi intermediate compartment (ERGIC) under the control of the small GTPases Rab1A, Rab1B, and Rab2A (Sun et al., 2024). The ERGIC has been proposed to play a role in autophagosome biogenesis through a non-classical type of COPII vesicle (Ge et al., 2013, 2017, 2014; Li et al., 2022). The THU pathway might thus cooperate with the autophagosome/endolysosomal compartments to achieve secretion. The THU pathway appears to be dependent on cytosolic cargo unfolding and is promoted by HSP90 chaperones (Zhang et al., 2020). More recently, studies have also demonstrated the involvement of TMED10-mediated UcPS in the secretion of IL-33, which plays a role in regulating intestinal epithelial differentiation and central nervous system homeostasis (Wang et al., 2024; Jiao et al., 2024). Another study reported that the UcPS of mutant huntingtin (Htt) is a multistep process including (1) the incorporation of Htt into the ERGIC by TMED10; (2) the transport of Htt through the autophagic pathway; and (3) its targeting to lysosomes, which then undergo exocytosis, leading to the release of Htt into the extracellular space (Ahat et al., 2022). In this series of events, GRASP55, a critical factor involved in various UcPS pathways (Bruns et al., 2011; Chiritoiu et al., 2019), appears to be essential in stabilizing the TMED10 channel and facilitating the autophagosome–lysosome fusion (Ahat et al., 2022). While TMED10 appears to play a critical role in the transport of cytosolic proteins into membrane intermediates during UcPS, structural analyses and cell-free reconstitution approaches are required to unambiguously consider TMED10 as a translocation channel. In addition to cargo proteins trafficked through the THU pathway, fatty acid binding protein-4 (FABP4) has recently been added to the growing list of cytosolic proteins that undergo UcPS. FABP4 secretion by adipocytes is independent of autophagosome and exosome biogenesis but requires sequential trafficking through endosomes and lysosomes (Villeneuve et al., 2018). However, the mechanisms and machinery involved in FABP4 translocation from the cytosol to the endosomal lumen remain to be characterized. Lastly, studies have reported that cytosolic HSP70, aldo-keto reductase family-1-member B8 (AKR1B8), and AKR1B10 can be released into the extracellular milieu after their translocation from the cytosol to LE/lysosomes via ABC transporters. Pharmacological inhibition of these transporters using glibenclamide, a non-specific ABC transporter inhibitor, or more selective inhibitors such as 4,4′-diisothiocyanostilbene-2,2′-disulfonic acid and bromosulfalein, resulted in a reduction of HSP70, AKR1B8, and AKR1B10 secretion, and of the release of markers of lysosomal exocytosis (Andrei et al., 1999; Mambula and Calderwood, 2006; Luo et al., 2011; Tang et al., 2014).

Collectively, these studies support the concept that in type III UcPS, cargo proteins can be incorporated into different intracellular compartments either by membrane remodeling and engulfment of the cytosol, as observed in autophagy and during the formation of intraluminal vesicles, or by protein channel-mediated translocation, as exemplified by pathways such as CMA, MAPS, or THU (Chiritoiu-Butnaru et al., 2022; Filaquier et al., 2022). Although several membrane-bound compartments appear to fulfill the role of vesicular intermediates capable of fusing with the PM in UcPS, a predominant point of convergence for the diverse routes recruiting cytosolic cargo proteins appears to be the LE/lysosomes (Fig. 2 B), highly dynamic organelles with exocytosis properties.

## Mechanisms and regulatory elements that control lysosome dynamics and exocytosis

Lysosomes, which range from 50 to 200 per cell, exhibit a non-uniform distribution throughout the cytosol. They also display significant heterogeneity in terms of dynamics, size, shape, intraluminal pH, and interactions with other intracellular compartments (Ballabio and Bonifacino, 2020). Furthermore, lysosomes are positioned at the crossroads of various trafficking pathways responsible for the transport of diverse substrates/cargoes, as well as specific structural and functional factors. Taken together, this implies that distinct lysosomal subpopulations may be specialized for specific functions, with their proportions dynamically adjusted in response to metabolic status, stress conditions, or external signals. In this context, a critical question arises: how can a degradative organelle be redirected for fusion with the PM for exocytosis? In other words, what makes a lysosome a secretory compartment? To answer succinctly, lysosomes must first relocate to the cell periphery, where they subsequently dock and fuse with the PM. This sequence of events involves numerous factors that induce profound changes in lysosomal properties.

### Lysosome positioning and motility

In most cell types, lysosomes are predominantly concentrated in the perinuclear region, near the Golgi apparatus, and around the microtube-organizing center. This positioning is orchestrated by retrograde lysosomal transport along microtubules, a process mediated by dynein motor proteins associated with dynactin (Burkhardt et al., 1997). The recruitment of dynein–dynactin to lysosomes involves the small GTPase Rab7, under the control of the GTPase-activating proteins TBC1D15 and TBC1D2, along with the guanine-nucleotide-exchange factor complex Mon1–Ccz1 (Frasa et al., 2010; Zhang et al., 2005; Nordmann et al., 2010). The Rab7-interacting lysosomal protein (RILP) and the cholesterol sensor OSBP-related protein 1L (ORP1L) have also been implicated in lysosome coupling to dynein–dynactin (Johansson et al., 2007; Progida et al., 2007). Conversely, the relocation of lysosomes to the cell periphery, a prerequisite for their exocytosis, is facilitated by anterograde lysosomal transport along microtubules via kinesin motor proteins (Hirokawa and Noda, 2008). Several kinesins, including the kinesin-1 proteins KIF5A, KIF5B, and KIF5C; the kinesin-2 protein KIF3; the kinesin-3 proteins KIF1A and KIF1B; and the kinesin-13 protein KIF2, participate in the anterograde transport of lysosomes (Pu et al., 2016). Kinesins interact directly with microtubules via a globular motor domain and with specific adaptors and cargos via a tail domain, facilitating their lysosomal binding and tracking. The mechanisms that couple kinesins to lysosomes are well characterized for specific kinesins. For example, kinesin-1 are recruited to lysosomes by the cooperative interaction of the multisubunit BLOC-1–related complex, the small GTPase Arl8 and its effectors Sif1, and the kinesin-interacting protein PLEKHM2 (Balderhaar and Ungermann, 2013; Pu et al., 2015). In addition, a variety of regulatory factors, including several Rab proteins, transmembrane lysosomal proteins such as LAMP1, LAMP2, or TMEM106B, intricately control the spatial distribution and bidirectional movement of lysosomes in conjunction with various factors or processes such as Ca^2+^ efflux from lysosomes, lipid composition of their membrane, intraluminal pH, organelle contact, and the actin cytoskeleton (Pu et al., 2016; Tancini et al., 2020; Buratta et al., 2020). Lysosome positioning and mobility within the cytoplasm are finely regulated in response to various perturbations, including changes in the metabolic status of the cell. Under conditions of nutrient depletion, lysosomes tend to cluster near the nucleus, facilitating fusion with autophagosomes, whereas nutrient availability promotes lysosomal movement toward the cell periphery (Korolchuk et al., 2011; Pu et al., 2016). In addition, in the context of proteotoxic stress, such as the accumulation of aggregation-prone proteins like α-synuclein, lysosomes containing these proteins relocate to the cell periphery, thereby becoming less acidic and losing their degradative properties (Dilsizoglu Senol et al., 2021).

### Lysosomal intraluminal pH

Lysosomal intraluminal pH is a critical parameter that profoundly affects lysosomal properties and exocytosis. Lysosomes are characterized by a highly acidic pH (4.5–5.0), which is optimal for the activity of most luminal lysosomal hydrolases involved in the degradation of various macromolecules. Interestingly, the cellular location of lysosomes has been shown to determine their intraluminal pH, with peripheral lysosomes exhibiting lower acidity and reduced degradative capacity (Johnson et al., 2016), potentially facilitating exocytosis (Andrei et al., 1999; Tapper and Sundler, 1995; Miao et al., 2015), compared with those located in the perinuclear region, which are more acidic and have higher degradative activity (Johnson et al., 2016). A possible explanation for this heterogeneity in intraluminal pH may reflect the stabilization of the vacuolar H+ ATPase (V-ATPase) by RILP, the Rab7-interacting lysosomal protein, which promotes lysosome location in the perinuclear region. Consequently, RILP contributes to the maintenance of acidic pH in juxtanuclear lysosomes compared with peripheral lysosomes (Johnson et al., 2016). Accordingly, inhibition of lysosomal acidification by pharmacological agents such as bafilomycin A1, a lysosomal V-ATPase inhibitor, stimulates lysosome fusion with the PM and the subsequent release of their contents into the extracellular milieu (Tapper and Sundler, 1995). Likewise, disruption of the lysosomal pH by lysosomotropic drugs such as ammonium chloride or chloroquine also increases the exocytosis of LE/lysosomes (Brown et al., 1985; Alvarez-Erviti et al., 2011). Collectively, these studies support the notion that a less acidic endolysosomal environment with compromised degradative activity is important for redirecting these organelles toward secretory function.

### Lysosome contacts with other intracellular compartments

In addition to their fusion with endosomes, autophagosomes, and the PM, lysosomes establish physical interactions through membrane contact sites (MCS) with various intracellular compartments, including the Golgi apparatus, the ER, mitochondria, and peroxisomes. These contacts/interactions play a pivotal role in sustaining interorganellar communication and profoundly influence lysosome properties, such as their intraluminal pH, motility, and positioning. For example, during nutrient starvation, the protein RILP bridges the lysosome-associated protein folliculin with the Golgi-associated small GTPase Rab34, promoting the perinuclear clustering of lysosomes (Starling et al., 2016), where lysosome–autophagosome fusion is facilitated (Korolchuk et al., 2011). The maintenance of the perinuclear population of endolysosomes is also facilitated by MCS between endolysosomes and the ER, mediated by the sorting nexin-19 (SNX19). SNX19, a transmembrane protein localized in the ER, tethers endolysosomes by interacting with the phosphoinositide PI(3)P on the endolysosomal membrane (Saric et al., 2021). In addition, PI(3)P associated with Rab7 on the endolysosomal membrane interacts with the ER-anchored protein protrudin (Raiborg et al., 2015). Other factors involved in ER–endolysosome interactions include RNF26, an ER-anchored ubiquitin ligase that facilitates the recruitment and ubiquitination of the cytosolic protein SQSTM1. Ubiquitinated SQSTM1 can then bind to the ubiquitin-binding domain of the adaptor proteins EPS15 and TAX1BP1, which are localized to the endolysosomal surface (Jongsma et al., 2016). VAMP-associated protein (VAP) is another ER-tethering factor, which interacts with the endolysosomal cholesterol-sensing protein ORP1L (Rocha et al., 2009). In addition to regulating lysosome motility and positioning, these interactions with ER membranes allow Ca2^+^ delivery from the ER to lysosomes, a process mediated by the clustering of inositol 1,4,5-triphosphate receptors at the ER–lysosome MCS (Atakpa et al., 2018). They also facilitate the non-vesicular transfer of lipids, such as phospholipids and cholesterol, between these organelles (Luo et al., 2017; Kumar et al., 2018). Lysosome–mitochondria and lysosome–peroxisome MCS are also critical in regulating the transfer of metabolites between these organelles and modulating lysosome dynamics (Leisten et al., 2023; Cisneros et al., 2022; Jin et al., 2015).

### Lysosome fusion with the PM

Under various stress conditions or in response to specific cellular demands, a subset of lysosomes with reduced acidity and degradative capacity, located proximal to the PM or translocated to the cell periphery, or lacking MCS with other intracellular compartments, can be mobilized for exocytosis. This process, which relies on the docking and fusion of lysosomes with the PM, is orchestrated by specific factors and specialized fusion machinery.

The docking of lysosomes to the outer leaflet of the PM occurs through the formation of a trans-SNARE complex involving the vesicle (v)-SNARE VAMP7 located on the lysosomal surface, and the target (t)-SNARE syntaxin-4 and synaptosome-associated protein of 23 kDa on the PM (Proux-Gillardeaux et al., 2007; Rao et al., 2004). Subsequently, lysosome–PM fusion is triggered by a local increase in intracellular Ca^2+^ levels, facilitated by the lysosomes themselves, which serve as Ca^2+^ reservoirs. Calcium efflux from lysosomes is mediated by the membrane protein mucolipin 1 (MCOLN1 or transient receptor potential mucolipin 1), which acts as a lysosomal Ca^2+^ channel. Calcium release from lysosomes binds to the Ca^2+^ sensor synaptotagmin VII (sytVII), which contains two C2 Ca^2+^-binding domains. This binding promotes the association of sytVII with preassembled trans-SNARE complexes and PM phospholipids, thereby facilitating lysosome fusion with the PM (Martinez et al., 2000). Interestingly, oxidative stress and reactive oxygen species have been shown to stimulate MCOLN1 activation, thereby facilitating Ca^2+^ release from lysosomes and subsequent exocytosis (Funato et al., 2020). Key regulators of this process include the small GTPases Rab3a and Rab10, which coordinate lysosomal docking at the PM for secretion (Vieira, 2018). In an inflammatory context, cytokines such as TNF-α promote lysosomal exocytosis, thereby facilitating α-synuclein propagation, an effect mediated by the involvement of the small GTPase Rab27A (Bae et al., 2022). Additionally, lysosomal structural proteins such as LAMP1 play a key role in lysosomal exocytosis. Sialylation of the N-terminal domain of LAMP1 facing the lysosomal lumen is critical. Knockdown of the sialidase N-acetyl-α-neuraminidase (Neu1) in mice enhances lysosomal recruitment and docking at the PM. Silencing LAMP1 reverses this phenotype, underscoring the importance of Neu1-controlled sialylation of LAMP1 in lysosomal exocytosis (Yogalingam et al., 2008). A study demonstrating that glucosylceramide accumulation within lysosomes induced by β-glucocerebrosidase inhibition enhances lysosomal exocytosis (Lunghi et al., 2022) also suggests that the lipid composition of lysosomes is a key determinant of their exocytosis.

## Concluding remarks and future perspectives

Located at the crossroads of multiple intracellular trafficking pathways, lysosomes are emerging as versatile organelles involved in diverse cellular functions that are critical for maintaining cellular homeostasis and adapting to stress conditions (Perera and Zoncu, 2016). In addition to their role as regulatory hubs for signaling, metabolism, and quality control, lysosomes exhibit remarkable capabilities in exocytosis, which is essential for the release of various cargo proteins. Consequently, they also play essential roles in intercellular communication, PM repair, ECM remodeling, and clearance of harmful materials independent of degradative mechanisms (Tancini et al., 2020; Reddy et al., 2001; Villeneuve et al., 2018; Filaquier et al., 2022). Recent advances have expanded our understanding of how cargo proteins converge to lysosomes, underscoring the critical role of lysosomes as sorting stations for both conventional and unconventional secretion pathways (Tancini et al., 2020; Filaquier et al., 2022). Elucidating the intricate pathways and molecular players involved in these processes, along with the various cargoes trafficked through lysosomes and subsequently released via exocytosis, is of paramount importance. An important objective in the field of UcPS will also be to decipher how cargo proteins can be differentially targeted to specific or multiples UcPS pathways depending on the pathophysiological conditions, cellular stress, and cell types. The establishment of standard in vitro cell–based assays as well as relevant in vivo models will be crucial to address this important issue in the near future.

**Figure 3.**
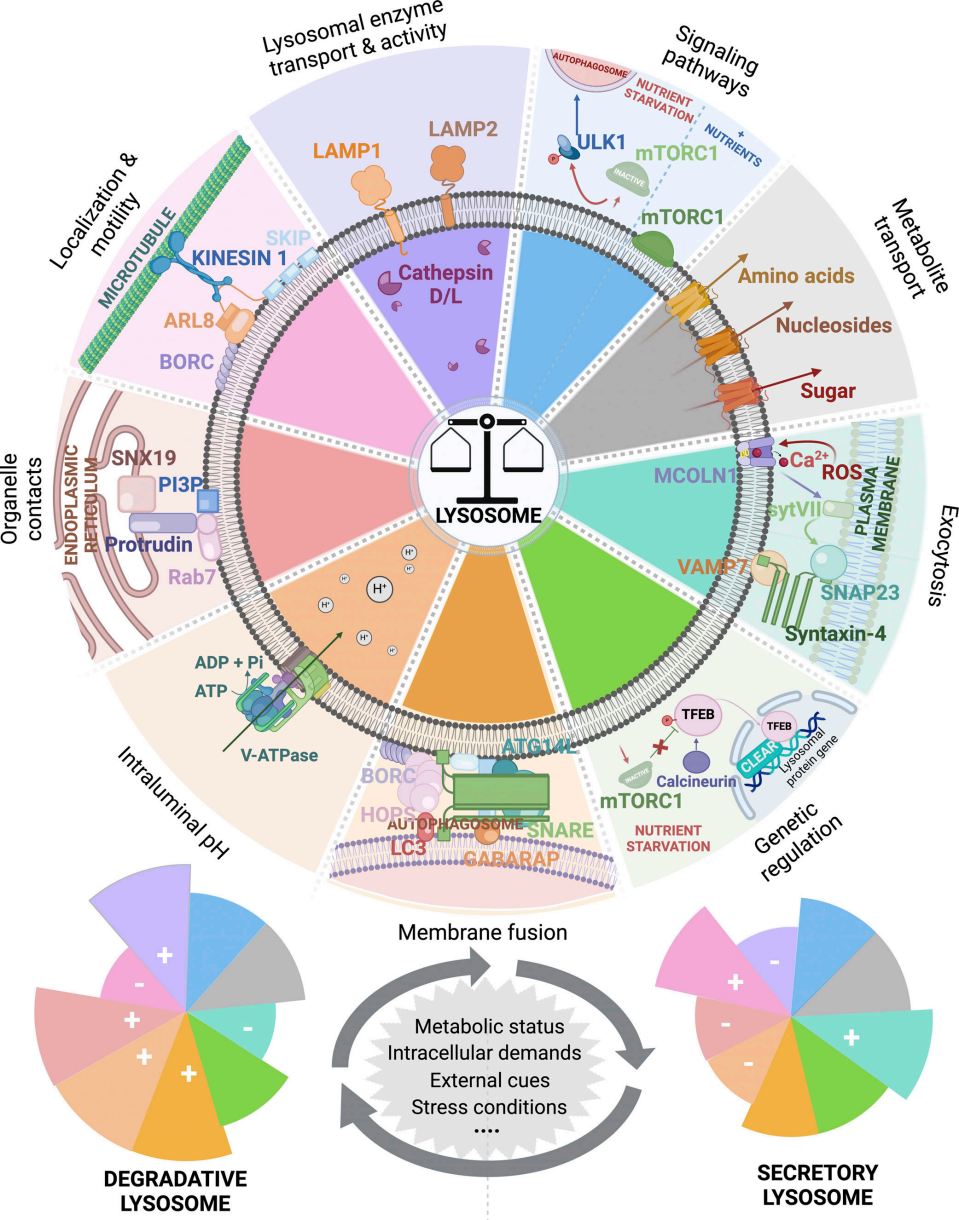
**Functional relationship between lysosomal properties.** Lysosomes are multifaceted organelles primarily known for their degradative properties sustained by structural and functional lysosomal proteins including, among others, LAMP1, LAMP2, and several hydrolases, such as Cathepsin D and L. The optimal activity of luminal hydrolyses is ensured by the acidic intraluminal pH of lysosomes (between 4.5 and 5.5) established by the vacuolar H^+^ APTase (v-ATPase). This facilitates the degradation and recycling of macromolecules, including proteins, nucleic acids, carbohydrates, and lipids, with the resulting metabolites exported to the cell through dedicated permeases. The transport into lysosomes of material from both intracellular and extracellular sources for degradation and recycling is allowed by the fusion of lysosomes with autophagosomes or LEs. Lysosomes also establish physical interactions with most of the others intracellular compartments through MCS. For instance, MCS between endolysosomes and the ER are mediated by the ER transmembrane protein SNX19 and the phosphoinositide PI(3)P located on the endolysosomal membrane. Lysosomes are also recognized as central hubs that sense nutrients and nucleic acids and from which different signaling pathways can be activated. Among the well-characterized autophagy regulators, the mTORC1 stands out as a master regulator of cell growth and metabolism. Under normal conditions, mTORC1 is activated on the lysosomal membrane (Dibble and Cantley, 2015) and negatively regulates autophagosome formation. However, during amino acid and glucose starvation, mTORC1 moves out of the lysosomes and become inactive (Son et al., 2019, 2020, 2024). This prevents the inhibition of the ULK1 complex and promotes autophagy (Hosokawa et al., 2009; Russell et al., 2013). The dissociation of mTORC1 from lysosomes also plays a critical role in the activation of a specific transcriptional program controlled by the transcription factor TFEB. In conditions associated to starvation or lysosome dysfunction, TFEB is translocated to the nucleus and binds to the CLEAR (coordinated lysosomal expression and regulation) motif presents on the promotor region of many genes encoding lysosomal enzymes and membrane proteins (Medina et al., 2011; Spampanato et al., 2013). Although lysosomes are distributed throughout the cytoplasm, they are mainly concentrated in the perinuclear area. Lysosome positioning and dynamics are orchestrated by transport along the microtubules, with the anterograde transport of lysosome from the perinuclear area to the cell periphery mediated by kinesin motor proteins. Once relocated to the cell periphery, lysosomes can fuse with the PM and release their content into the extracellular space. Lysosomal exocytosis is ensured by the mobilization of a specific fusion machinery and is triggered by an increase in intracellular Ca^2+^ concentration. Overall, while lysosomes are primarily known for their degradative function, in response to intrinsic demands, external cues, and environmental changes, lysosomal properties can be finely regulated and adapted, cooperating to convert lysosomes into secretory compartments. BORC, BLOC-1–related complex; HOPS, homotipic fusion and vacuole protein sorting. The figure was created with Biorender.

Lysosomal functions rely on coordinated processes involving the activation, recruitment, and transport of diverse molecular factors to lysosomes that shape their content, positioning, dynamics, interactions, and fusion with other organelles. The complex interplay between different lysosomal properties delineates their multifaceted roles as degradative, signaling, or secretory organelles. Importantly, these lysosomal properties are not mutually exclusive, but exhibit profound interdependencies that orchestrate lysosomal functions toward specific cellular outcomes. Different cellular demands or stress conditions can regulate or alter lysosomal functionality. For instance, disruptions in trafficking along the ER–Golgi secretory axis, as evidenced in pathological contexts such as cancer or pathogen infection (Banerjee et al., 2023; Chen et al., 2022), may affect the delivery of essential structural and functional components to lysosomes, including the V-ATPase or degradative enzymes. Similarly, the accumulation of excessive or deleterious materials within lysosomes, as observed in lysosomal storage disorders or neurodegenerative diseases can lead to lysosomal membrane permeabilization (Walkley and Vanier, 2009; Piovesana et al., 2023; Rose et al., 2023, *Preprint*), resulting in stressed lysosomes and a vicious cycle that compromises their intraluminal pH, catabolic properties, interactions with organelles, dynamics, and positioning. This can affect lysosomal content and induce lysosomal remodeling through the activation of both transcriptional and post-transcriptional programs (Perera and Zoncu, 2016; Zoncu and Perera, 2022). Thus, numerous interrelated events triggered in response to cellular nutritional status, stress conditions, or physiological demands differentially regulate lysosomal properties, potentially contributing to make a subset of secretion-competent lysosomes (Fig. 3). Therefore, unravelling the subtle interplay and functional relationship between lysosome properties and identifying molecular effectors and regulatory elements that act as molecular switches to convert lysosomes into secretory compartments are critical areas of investigation.

An important challenge is also to understand the mechanisms governing lysosomal heterogeneity within individual cells, and the mobilization of subpopulations of lysosomes for specific functions. It is worth noting that the lysosomal population includes autolysosomes, endolysosomes, and terminal/storage lysosomes, which coexist in a dynamic equilibrium intricately linked to the lysosomal regeneration cycle (Bright et al., 1997, 2016). During this cycle, terminal/storage lysosomes are reformed from autolysosomes and endolysosomes through processes such as tubulation, maturation, and content condensation (Yang and Wang, 2021). Remarkably, terminal/storage lysosomes exhibit reduced acidity and degradative activity compared with endolysosomes (Bright et al., 2016). Accordingly, the lysosomal regeneration cycle introduces an additional level of complexity to the heterogeneity of the lysosomal population, where nutritional, signaling, and stress cues can modulate lysosome properties to redirect this compartment toward secretion. Whether lysosomes undergoing secretion are also degradation competent, or whether the acquisition of secretory properties is detrimental to other lysosomal functions, remain important questions that warrant further exploration.

In addition to unraveling fundamental processes that govern lysosomal biology, the dissection of the molecular mechanisms that support the conversion of lysosomes into secretory organelles will also provide opportunities for the development of new therapeutic strategies in several diseases. Indeed, dysfunctional lysosomal degradation is a pathological hallmark of lysosomal storage disorders (Walkley and Vanier, 2009; Platt et al., 2018) and neurodegenerative diseases such as Parkinson’s and Alzheimer’s diseases (Fleming et al., 2022), which are characterized by the accumulation of harmful undigested substrates and misfolded proteins, respectively. Several studies have demonstrated that enhancing cellular clearance through lysosomal exocytosis can mitigate the deleterious accumulation of toxic material (Tsunemi et al., 2019; Xu et al., 2020; Medina et al., 2011; Spampanato et al., 2013). In these studies, lysosomal exocytosis was triggered either by a Ca^2+^ channel agonist (Tsunemi et al., 2019), or by activation of a specific transcriptional program controlled by the transcription factor EB (TFEB) (Xu et al., 2020; Medina et al., 2011; Spampanato et al., 2013), whose nuclear-cytoplasmic shuttling is regulated by mechanistic target of rapamycin complex 1 (mTORC1) (Roczniak-Ferguson et al., 2012; Settembre et al., 2012; Takla et al., 2023). TFEB orchestrates the transcriptional expression of numerous genes involved in autophagy and lysosome biogenesis (Martina et al., 2012; Settembre et al., 2011). It also upregulates the expression of the lysosomal Ca^2+^ channel MCOLN1, facilitating lysosome fusion with the PM and release of lysosomal contents into the extracellular milieu (Xu et al., 2020; Medina et al., 2011). However, the concept that the conversion of lysosomes into secretory compartments represents an alternative to degradation processes, particularly in neurodegenerative diseases, warrants careful consideration. Studies suggest that the release of toxic misfolded proteins such as Tau and α-synuclein, while beneficial to the cells in which they accumulate, may have detrimental effects at the organismal level, by promoting the propagation of toxic protein species and disease progression in Alzheimer’s and Parkinson’s diseases (Neupane et al., 2023; Dujardin et al., 2014; Clavaguera et al., 2009). The adverse consequences of material clearance via lysosomal exocytosis are also amplified by the dissemination of toxic misfolded proteins between neighboring cells through the transport of lysosomes via tunneling nanotubes (Dilsizoglu Senol et al., 2021; Abounit et al., 2016). Overall, this suggests that targeting molecular players involved in lysosome transport and exocytosis holds promise for the treatment of neurodegenerative diseases, although the development and validation of such strategies require in vivo models that faithfully recapitulate the dynamic spatiotemporal progression of lesions observed in patients.

In conclusion, there is no doubt that research in lysosome biology will unveil key mechanisms of intercellular communication and enhance our understanding of various disease conditions.
